# Gut microbiota-based machine-learning signature for the diagnosis of alcohol-associated and metabolic dysfunction-associated steatotic liver disease

**DOI:** 10.1038/s41598-024-60768-2

**Published:** 2024-07-12

**Authors:** In-gyu Park, Sang Jun Yoon, Sung-min Won, Ki-Kwang Oh, Ji Ye Hyun, Ki Tae Suk, Unjoo Lee

**Affiliations:** 1 Department of Electrical Engineering, Hallym University, Gyo-dong, Chuncheon, 24253 Republic of Korea; 2 Institute for Liver and Digestive Diseases, Hallym University, Chuncheon, 24253 Republic of Korea; 3 Department of Internal Medicine, Hallym University Chuncheon Sacred Heart Hospital, Hallym University, Gyo-dong, Chuncheon, 24253 Republic of Korea

**Keywords:** Alcoholic-associated liver disease, Metabolic dysfunction-associated steatotic liver disease, Microbiota, Machine learning, Diagnosis, Computational biology and bioinformatics, Microbiology, Gastroenterology

## Abstract

Alcoholic-associated liver disease (ALD) and metabolic dysfunction-associated steatotic liver disease (MASLD) show a high prevalence rate worldwide. As gut microbiota represents current state of ALD and MASLD via gut-liver axis, typical characteristics of gut microbiota can be used as a potential diagnostic marker in ALD and MASLD. Machine learning (ML) algorithms improve diagnostic performance in various diseases. Using gut microbiota-based ML algorithms, we evaluated the diagnostic index for ALD and MASLD. Fecal 16S rRNA sequencing data of 263 ALD (control, elevated liver enzyme [ELE], cirrhosis, and hepatocellular carcinoma [HCC]) and 201 MASLD (control and ELE) subjects were collected. For external validation, 126 ALD and 84 MASLD subjects were recruited. Four supervised ML algorithms (support vector machine, random forest, multilevel perceptron, and convolutional neural network) were used for classification with 20, 40, 60, and 80 features, in which three nonsupervised ML algorithms (independent component analysis, principal component analysis, linear discriminant analysis, and random projection) were used for feature reduction. A total of 52 combinations of ML algorithms for each pair of subgroups were performed with 60 hyperparameter variations and Stratified ShuffleSplit tenfold cross validation. The ML models of the convolutional neural network combined with principal component analysis achieved areas under the receiver operating characteristic curve (AUCs) > 0.90. In ALD, the diagnostic AUC values of the ML strategy (vs. control) were 0.94, 0.97, and 0.96 for ELE, cirrhosis, and liver cancer, respectively. The AUC value (vs. control) for MASLD (ELE) was 0.93. In the external validation, the AUC values of ALD and MASLD (vs control) were > 0.90 and 0.88, respectively. The gut microbiota-based ML strategy can be used for the diagnosis of ALD and MASLD.

ClinicalTrials.gov NCT04339725

## Introduction

Alcohol-associated liver disease (ALD) and metabolic dysfunction-associated steatotic liver disease (MASLD) are medical conditions that show a high mortality rate worldwide and they account for the majority of liver diseases (LD)s^1–3^. Pathologically, LD is characterized by steatosis, inflammation, fibrosis, hepatocellular carcinoma, and regeneration of the liver parenchyma. The progression of LD is now known to be a dynamic process with significant potential for resolution^4,5^. Therefore, early diagnosis is an essential part of the assessment and management of patients with chronic LD. Current diagnostic strategies range from the use of biomarkers to image modalities^6^. Classical diagnostic methods, such as biopsy and imaging studies, have disadvantages, such as high cost, need for hospitalization, inability to diagnose general health state, and side effects (bleeding, infection, pain, or mortality). As such, there is strong demand for reliable diagnostic biomarkers that provide insight into disease in lieu of invasive liver biopsy. Future personal medicine requires a simple and non-invasive method that can simultaneously identify and predict various health conditions in LD^7^.

The human gut microbiome is an ecosystem that consists of various microorganisms including bacteria, fungi, and viruses^8^. The gut microbiota plays multiple roles in human health related to the immune system, inflammation, nutritional balance, homeostasis, circulation, and metabolism. To date, the distribution of intestinal microorganisms is affected by several factors, such as genetics, nutrition, the environment, exercise, pathobiology, and lifestyle^9^. When the balance between the human and gut microbiota is disorganized, the composition and function of the microbiota are changed, which is referred to as dysbiosis^10^. Gut microbial imbalance is associated with the occurrence of various diseases, and each disease shows characteristic findings of intestinal microorganisms^11^. Because gut microbiota reflect an individual’s health and predicts prognosis, it has revealed promising biomarker for various diseases^12^. In this respect, the stool microbiota analysis-based diagnostic strategy has the advantage of being able to diagnose chronic LD quickly and accurately at a relatively low cost.

The gut–liver axis refers to the close bidirectional connection between the intestine and the liver via the portal vein, biliary tract, and systemic circulation. There has been increasing interest in the role of the gut microbiome in LD as a potential diagnostic modality, therapy, or prognostic marker^13,14^. Gut microbiota dysregulation plays a key role in the pathogenesis of LD through the gut–liver axis. It is difficult to identify their characteristics and rules because individual differences and diversity exist. The intestinal microbiota, which reflects an individual's health status, has a close relationship with LDs. Recent studies have demonstrated that the composition of the gut microbiome differs according to the progression of LD^15,16^. Accordingly, LD can be diagnosed using the distribution of the gut microbiome profiles^17^.

It is necessary to apply diagnostic technology that considers various factors using artificial intelligence to achieve accurate and personalized medical treatment. Machine learning (ML), one branch of artificial intelligence, has been utilized for the diagnosis of various diseases, including malignancy, metabolic disease, and intestinal disease^18^. Recently, artificial intelligence has been used in the diagnosis of LD. Most ML studies analyzed genetic information or clinical data^19,20^. Few ML studies have utilized gut microbiota in the diagnosis of LD, and data covering various stages of LD are insufficient. Currently, the ML technique is not applied in the diagnosis of ALD. Considering that LD progresses sequentially and that the microbiota is closely related to LD, we hypothesized that a gut microbiota-based ML strategy could be used for the diagnosis of ALD and MASLD.

## Results

### Study population

**Figure 1:**
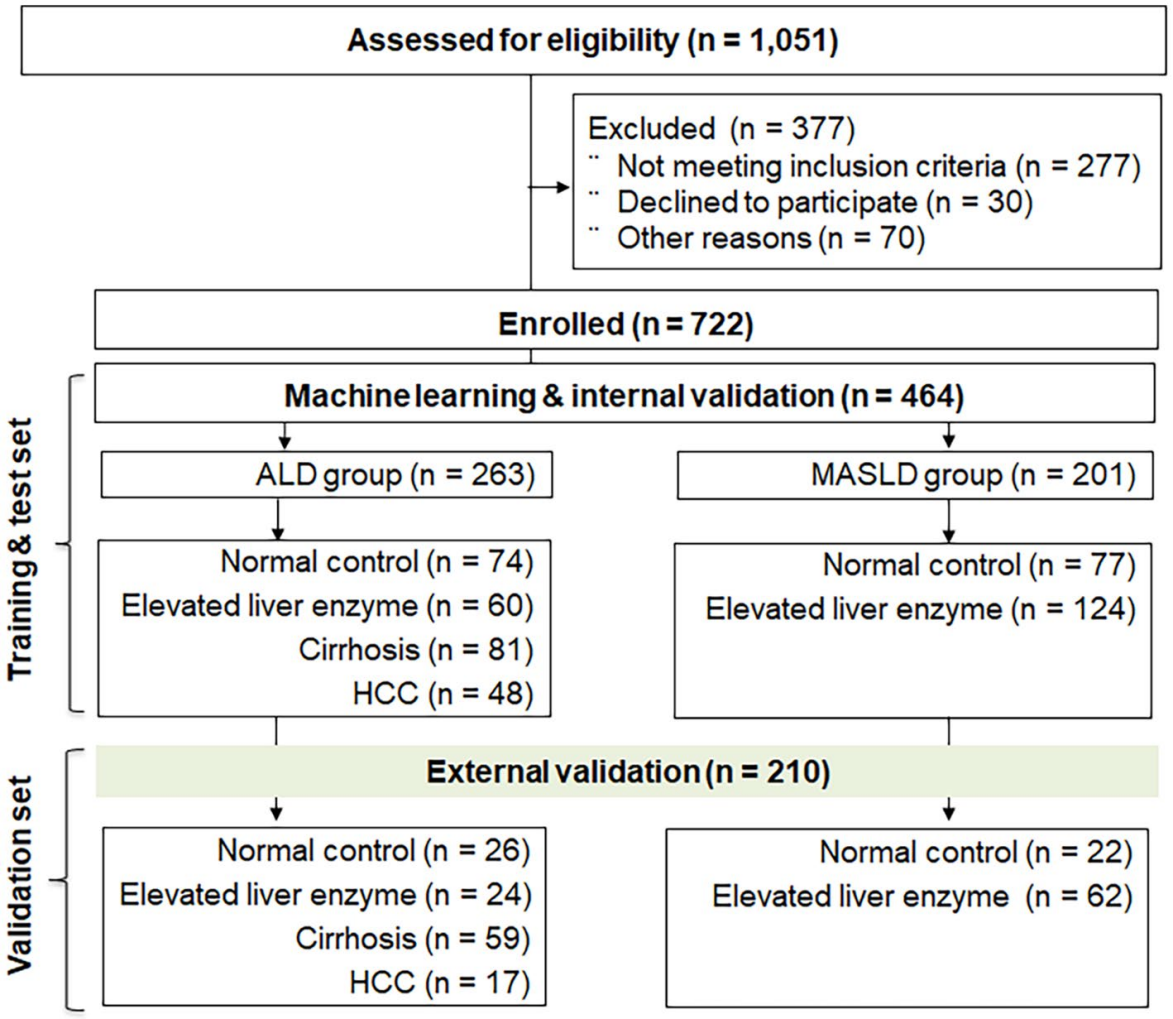
Study design.

**Figure 2:**
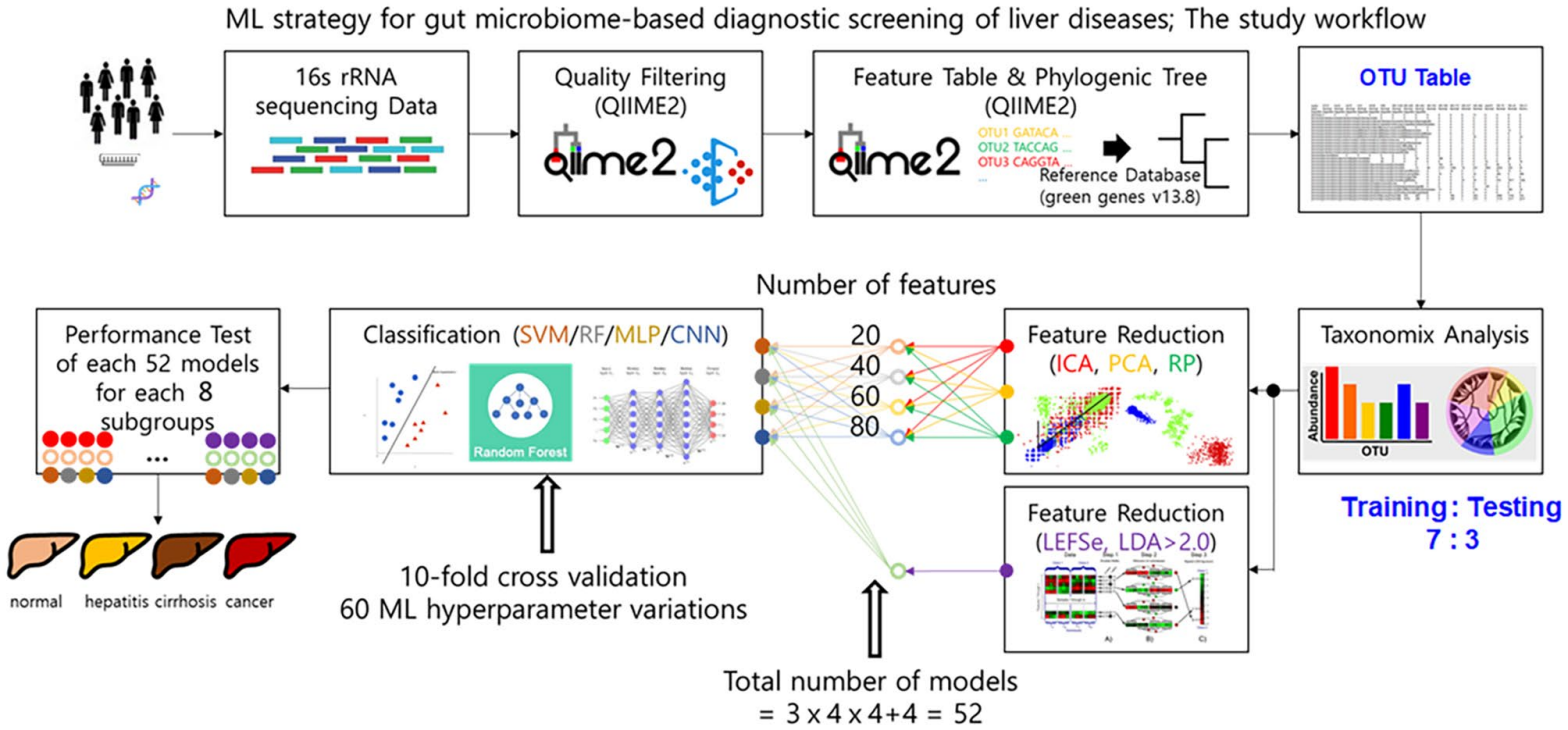
Machine learning strategy.

A prospective cohort study was performed between April 2017 and March 2020 (ClinicalTrials.gov NCT04339725). This study involved patients with LD who had follow-up visits at the hepatology department of University Hospitals. A total of 1,051 people underwent stool analysis for metagenomics by sequencing the 16S rRNA gene (Fig. 1). The workflow of the whole study is summarized in Fig. 2. Detailed baseline characteristics are explained in Table S1.

### Microbial differences in alcohol-associated liver disease

**Figure 3:**
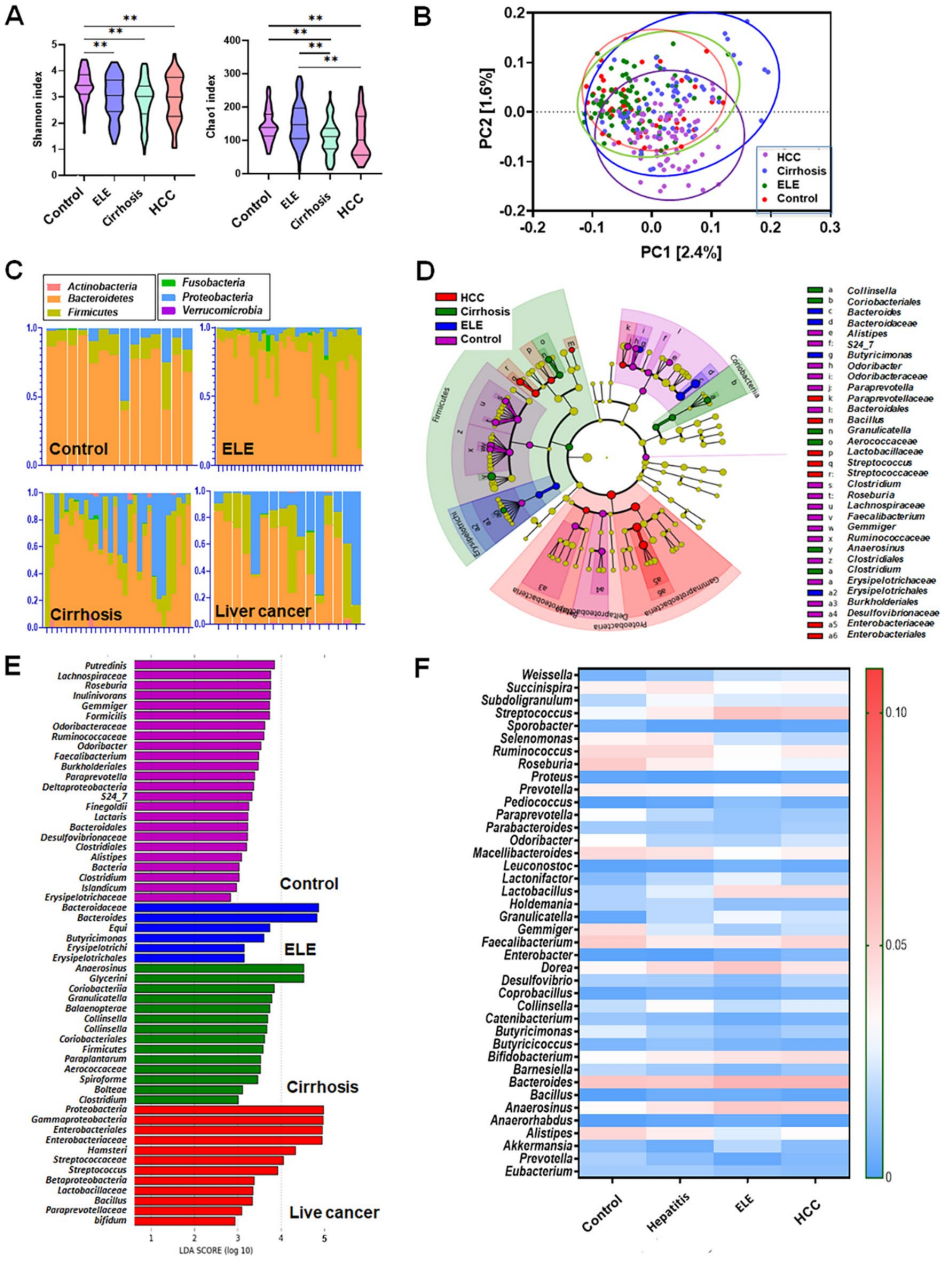
(**A**) Differences in alcoholic liver disease group. (**B**) α diversity. β diversity. (**C**) Composition of phylum. (**D**) Taxonomical features with a LDA score > 2.0 were plotted with cladogram and (**E**) LEfSe bar graph for each group. (**F**) Heatmap for different genus and species. *ELE* elevated liver enzyme; *HCC* hepatocellular carcinoma.

The control group revealed higher Shannon index scores than the elevated liver enzyme (ELE), cirrhosis, and liver cancer groups (*p* < 0.01). No difference in Shannon’s index was found among ELE, cirrhosis, and liver cancer. This result is also shown in the inverse Simpson index and Plelou evenness (Fig. S1A). The Chao1 index of the control and ELE groups was higher than that of the alcoholic cirrhosis and liver cancer groups (*p* < 0.01). The ELE group showed a difference compared with the cirrhosis and liver cancer groups in the Chao1 index (*p* < 0.01) (Fig. 3A).

In the β diversity analysis and composition of phyla, each group showed differences (Fig. 3B,C). β diversity ordination using the Aitchison distance: applying PCA to the centered log-ratio (CLR) transformed counts. By using unweighted UniFrac distance, PCoA analysis indicated the different community compositions at the OTU level between the control and the liver fibrosis groups with variances of PC1 1.6% and PC2 2.4%.

There was a difference in the centroid location between groups (Pr [> F] = 0.001) (Fig. S1B). LEfSe analysis was performed to identify the distinct bacterial species between the control and ALD groups with an LAD score > 2 (Table S2 and S4, Fig. 3D,E).

In the liver cancer group, the levels of *Proteobacteria* (phylum), *Betaproteobacteria* and *Gammaproteobacteria* (class), *Enterobacteriales* (order), *Enterobacteriaceae, Streptococcaceae,* and *Paraprevotellaceae* (family), *Lactobacillus*, *Streptococcus*, and *Bacillus* (genus) (*p* < 0.05), and *Bifidum* and *Hamsteri* (species) were found to be higher.

Regarding cirrhosis, *Firmicutes*, *Coriobacteriia*, *Coriobacteriales, Aerococcaceae, Collinsella, Granulicatella, Anaerosinus, Clostridium, Balaenopterae, Paraplantarum, Bolteae, Glycerini,* and spiroforme were found to be increased (*p* < 0.005). The abundances of *Erysipelotrichim, Erysipelotrichales, Bacteroidaceae, Butyricimonas, Bacteroides,* and *equi* were increased in the alcoholic ELE group (*p* < 0.0005). *Deltaproteobacteria, Bacteroidales, Clostridiales, Burkholderiales, Odoribacteraceae*, S24_7*, Lachnospiraceae, Ruminococcaceae, Erysipelotrichaceae, Desulfovibrionaceae, Odoribacter, Paraprevotella, Alistipes, Clostridium, Roseburia, Faecalibacterium, Gemmiger, Finegoldii, Putredinis, Inulinivorans, Lactaris, Islandicum,* and *Formicilis* were significantly increased in the control group (Fig. 3F). Circos representation of the most abundant bacterial genera between the normal, ELE, cirrhosis, and cancer groups in the ALD datasets. Bacterial genera with LDA scores above 2.0 for all bacterial sequences were plotted (Fig. S1C).

### Microbial differences in metabolic dysfunction-associated steatotic liver disease

**Figure 4:**
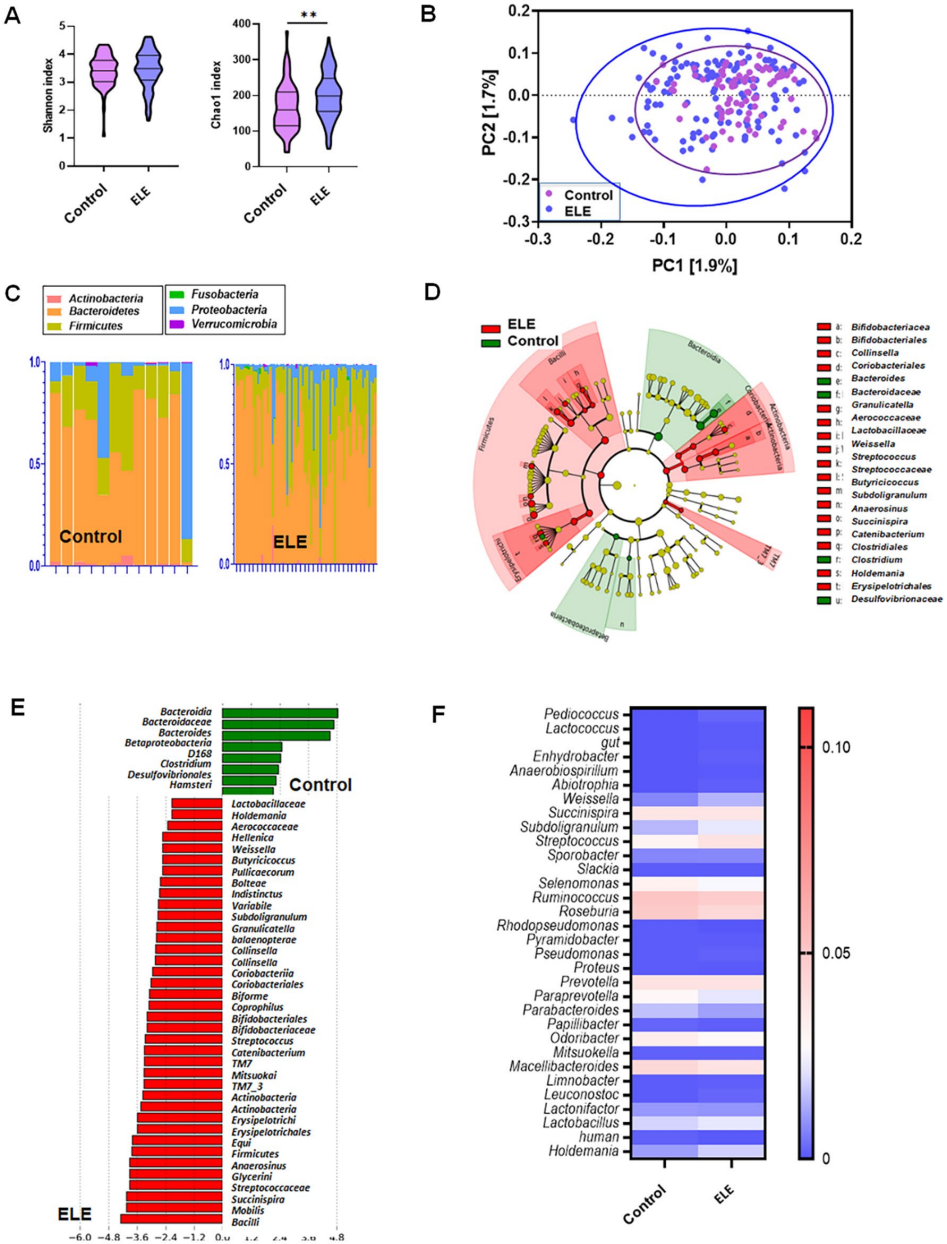
(**A**) Differences in metabolic dysfunction-associated steatotic liver disease group. (**B**) α diversity. β diversity. (**C**) Composition of phylum. (**D**) Taxonomical features with a LDA score > 2.0 were plotted with cladogram and (**E**) LEfSe bar graph for each group. (**F**) Heatmap for different genus and species. *ELE* elevated liver enzyme.

No differences in Shannon (*p* = 0.339) or inverse Simpson (*p* = 0.401) indices were found between the control and ELE groups (Fig. 4A and Fig. S2A). The Chao1 index of the α diversity in the control group was lower than that in the ELE group (*p* = 0.002). PCoA indicated the different community compositions at the OTU level between the control group and the liver fibrosis group, with variances of PC1 of 1.9% and PC2 of 1.7% (Fig. 4B and Fig. S2B).

In the ELE group, significantly increased relative abundances of *Actinobacteria, Firmicutes,* and TM7 were found at the phylum level (*p* < 0.05). At the class level, higher abundances of *Actinobacteria, Coriobacteriia, Bacilli, Erysipelotrichia,* and TM7_3 were found (*p* < 0.05). At the species level, a higher abundance of *Coprophilus, Indistinctus, Balaenopterae, Hellenica, Equi, Bolteae, Pullicaecorum, Variabile, Glycerini, Mobilis, Biforme,* and *Mitsuokai* was found in the ELE group (*p* < 0.005).

In the control group, a lower abundance of *Bacteroidia* and *Betaproteobacteria* was found at the class level (*p* < 0.05). At the order level, *Desulfovibrionales* decreased significantly in the control group (*p* < 0.05). At the family level, a lower abundance of *Bacteroidaceae* was identified, and *Bacteroides* decreased significantly at the genus level (*p* < 0.05). At the species level, lower abundances of *Hamsteri, Clostridium,* and D168 were found in the control group (*p* < 0.05) (Fig. 4C–F). Circos representation of the most abundant bacterial genera between the normal and ELE groups in MASLD datasets. Bacterial genera with LDA scores above 2.0 for all bacterial sequences were plotted. (Table S3, S4 and Fig. S2C).

### Diagnostic value of supervised machine learning models and external validation

Table 1 presents performance measures of the four different ML algorithms evaluated on each of the testing datasets of ALD and MASLD. Here, the 40 reduced feature dimensions are considered for both the ALD and MASLD groups. CNN in classification combined with PCA in feature reduction performed better than other models. They achieved AUC values ranging between 0.92 and 0.96 for ALD datasets and an AUC of ≈ 0.96 for an MASLD dataset. Tables present performance measures of the CNN model for four different numbers (20, 40, 60 and 80) of feature dimensions reduced by the PCA model for the ALD and MASLD datasets (Tables S4 and S5, respectively) as well as the architecture of the CNN model utilized in this study (Table S6).

**Table 1:** Performance measures of different machine learning algorithms.

	Group 1	Group 2	AUC				
				SVM	RF	MLP	CNN
ALD	Normal control	ELE	ICA	0.80 ± 0.08	0.7 ± 0.08	0.71 ± 0.06	0.93 ± 0.11
			PCA	0.86 ± 0.05	0.82 ± 0.06	0.76 ± 0.06	0.94 ± 0.08
			RP	0.86 ± 0.04	0.83 ± 0.04	0.77 ± 0.08	0.94 ± 0.07
			LDA > 2.0	0.52 ± 0.02	0.48 ± 0.06	0.54 ± 0.11	0.86 ± 0.15
		Cirrhosis	ICA	0.92 ± 0.03	0.77 ± 0.04	0.91 ± 0.04	0.97 ± 0.03
			PCA	0.95 ± 0.02	0.92 ± 0.02	0.92 ± 0.02	0.96 ± 0.07
			RP	0.94 ± 0.03	0.91 ± 0.03	0.92 ± 0.03	0.97 ± 0.04
			LDA > 2.0	0.54 ± 0.06	0.69 ± 0.05	0.55 ± 0.06	0.87 ± 0.15
		HCC	ICA	0.92 ± 0.04	0.82 ± 0.04	0.86 ± 0.06	0.95 ± 0.10
			PCA	0.96 ± 0.03	0.91 ± 0.05	0.92 ± 0.07	0.96 ± 0.08
			RP	0.92 ± 0.05	0.91 ± 0.06	0.92 ± 0.03	0.96 ± 0.05
			LDA > 2.0	0.66 ± 0.07	0.80 ± 0.05	0.62 ± 0.09	0.88 ± 0.13
	Normal control + ELE	Cirrhosis + HCC	ICA	0.86 ± 0.04	0.78 ± 0.04	0.81 ± 0.05	0.91 ± 0.10
			PCA	0.88 ± 0.03	0.86 ± 0.03	0.84 ± 0.04	0.96 ± 0.07
			RP	0.86 ± 0.04	0.84 ± 0.04	0.83 ± 0.05	0.89 ± 0.09
			LDA > 2.0	0.53 ± 0.04	0.67 ± 0.04	0.55 ± 0.05	0.89 ± 0.13
	ELE	Cirrhosis	ICA	0.8 ± 0.07	0.67 ± 0.09	0.78 ± 0.08	0.90 ± 0.13
			PCA	0.86 ± 0.04	0.76 ± 0.08	0.81 ± 0.06	0.94 ± 0.12
			RP	0.86 ± 0.04	0.83 ± 0.06	0.81 ± 0.04	0.85 ± 0.07
			LDA > 2.0	0.54 ± 0.06	0.52 ± 0.08	0.55 ± 0.09	0.84 ± 0.17
		HCC	ICA	0.82 ± 0.05	0.59 ± 0.12	0.76 ± 0.05	0.91 ± 0.12
			PCA	0.88 ± 0.05	0.84 ± 0.06	0.85 ± 0.07	0.96 ± 0.07
			RP	0.79 ± 0.07	0.86 ± 0.05	0.87 ± 0.05	0.95 ± 0.08
			LDA > 2.0	0.59 ± 0.08	0.59 ± 0.06	0.59 ± 0.10	0.88 ± 0.14
	Cirrhosis	HCC	ICA	0.69 ± 0.08	0.52 ± 0.09	0.68 ± 0.08	0.87 ± 0.16
			PCA	0.77 ± 0.10	0.72 ± 0.05	0.71 ± 0.07	0.93 ± 0.11
			RP	0.76 ± 0.08	0.69 ± 0.05	0.73 ± 0.11	0.87 ± 0.12
			LDA > 2.0	0.60 ± 0.12	0.54 ± 0.10	0.55 ± 0.10	0.88 ± 0.16
MASLD	Normal	ELE	ICA	0.72 ± 0.06	0.68 ± 0.07	0.73 ± 0.05	0.87 ± 0.11
			PCA	0.77 ± 0.05	0.74 ± 0.04	0.74 ± 0.08	0.93 ± 0.11
			RP	0.76 ± 0.05	0.77 ± 0.06	0.72 ± 0.07	0.88 ± 0.10
			LDA > 2.0	0.56 ± 0.06	0.62 ± 0.07	0.60 ± 0.07	0.85 ± 0.16

*ELE* elevated liver enzyme; *HCC* hepatocellular carcinoma; *ICA* independent component analysis; *PCA* principal component analysis; *RP* random projection; *SVM* support vector machine; *RF* random forest; *MLP* multilevel perceptron; *CNN* convolutional neural network.

Forty reduced feature dimensions are considered from each of ICA, PCA, and RP.

**Figure 5:**
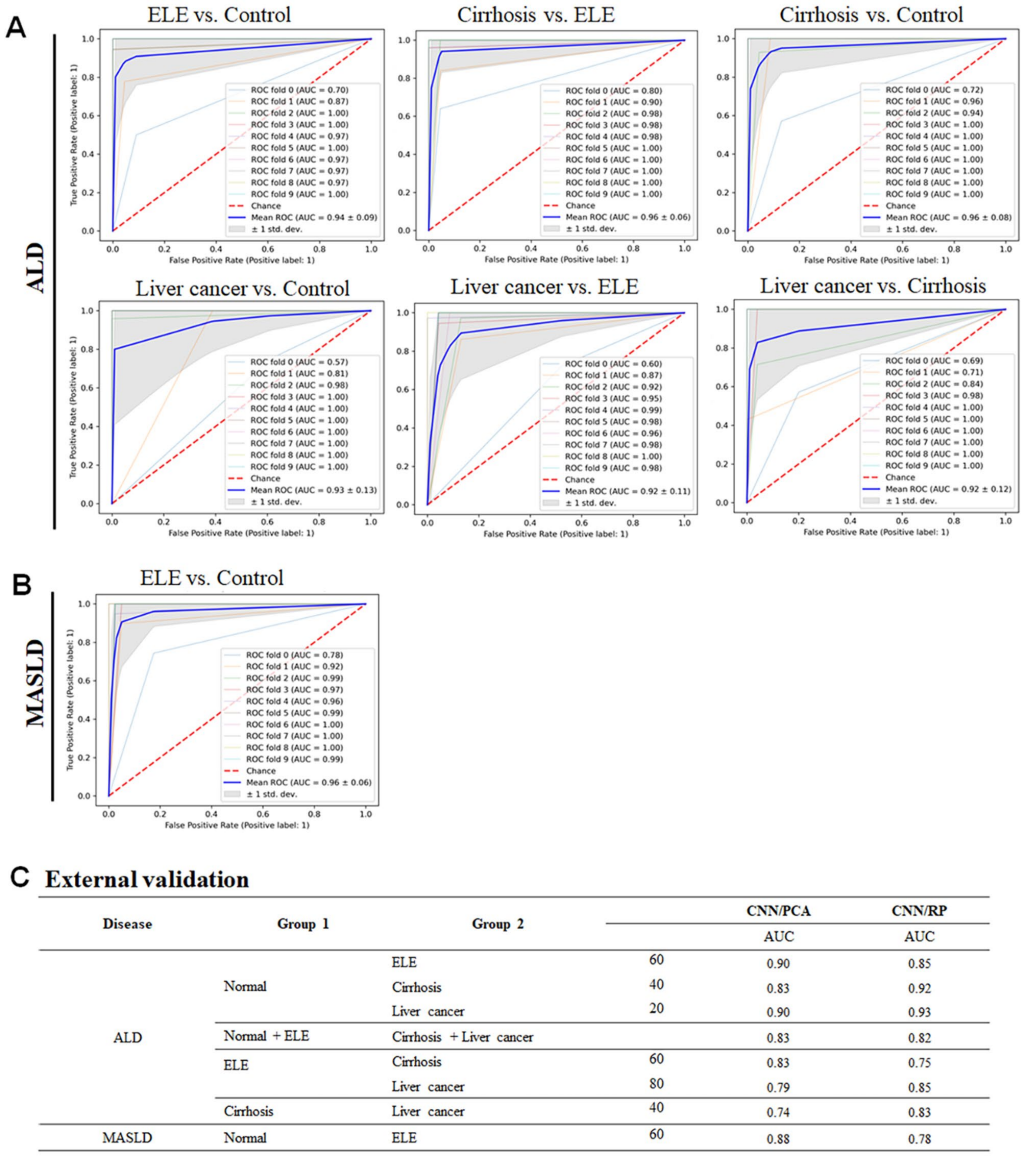
(**A**) Diagnostic values of machine learning strategy in liver disease. Alcoholic liver disease. (**B**) Metabolic dysfunction-associated steatotic liver disease. (**C**) External validation. *ELE* elevated liver enzyme; *HCC* hepatocellular carcinoma.

With the ALD datasets, precision, recall, and accuracy were in the range of 0.89 to 0.96, 0.94 to 0.99, and 0.94 to 0.98, respectively, for the 20 reduced feature dimensions; in the range of 0.91 to 0.96, 0.95 to 0.99, and 0.95 to 0.97, respectively, for the 40 reduced feature dimensions; in the range of 0.88 to 0.96, 0.94 to 0.98, and 0.94 to 0.98, respectively, for the 60 reduced feature dimensions; and in the range of 0.88 to 0.97, 0.94 to 0.99, and 0.93 to 0.98, respectively, for the 80 reduced feature dimensions (Fig. 5A). With the MASLD datasets of the normal and ELE groups, precision, recall, and accuracy were 0.94, 0.93, and 0.92, respectively, for the 20 reduced feature dimensions; 0.95, 0.95, and 0.94, respectively, for the 40 reduced feature dimensions; 0.97, 0.95, and 0.95, respectively, for the 60 reduced feature dimensions; and 0.98, 0.96, and 0.96, respectively, for the 80 reduced feature dimensions (Fig. 5B). The CNN model trained with the 40 reduced feature dimensions had slightly higher sensitivity and specificity, showing good diagnostic classification power for predicting and identifying the disease groups.

In the external validation, the AUC values for ALD and MASLD (vs control) were > 0.90 and 0.88, respectively, considering the higher values between the combinations CNN/PCA and CNN/RP (Fig. 5C).

## Discussion

In LD, artificial intelligence has been applied for detecting fibrosis, differentiating liver mass, predicting the prognosis of chronic LD, and diagnosing MASLD^21^. In a previous report, the development and verification of ML and artificial intelligence using gut microbiome data for cancer treatment and diagnosis was evaluated^22^. Loomba et al*.* provided a random forest classifier mode that showed excellent diagnostic accuracy in detecting advanced fibrosis in MASLD (AUC 0.936)^23^. In our clinical data for ALD and MASLD, ML models of convolutional neural networks combined with principal component analysis with 40 taxonomic features achieved > 0.90 in the AUC scores on the 8 paired groups, showing the potential of using ML for predictive diagnosis. Compared with other studies utilizing the microbiome, our current data first link metagenomic features with ALD and MASLD, which leads to the discovery of a potential ML model for diagnosing ALD and MASLD.

In the external validation results using patient data from different regions, the AUC was over 0.90 for ALD and 0.88 for MASLD. These results show that our ML strategy can be useful for diagnosing ALD and MASLD.

As typical microbiota signatures affect the development and progression of human diseases, demonstration of the relationship between the gut microbiome and disease characteristics might be the cornerstone in future human health care^24,25^. With advances in microbiome-related technologies and personalized medicine, the vast amount of data and the complexity of the data limit statistical analysis and predictive potential. Considering the diversity of gut microbiota due to multiple factors, universally applicable microbiota-based metagenomic signatures are not known^26^. Recently, ML and artificial intelligence technology have been actively applied to the analysis of large-scale health care information and the utilization of microbiome-based big datasets, and application cases are being reported in various diseases^27^.

In the ALD, specific AUCs for the diagnosis of liver cirrhosis reached 0.97, indicating that alcoholic cirrhosis patients showed a typical dysbiosis pattern compared with the pattern of the control or ELE group. *Anaerosinus, Glycerini, Coriobacteriia, Granulicatella*, *Balaenopterae*, *Collinsella*, *Collinsella*, *Coriobacteriales*, *Paraplantarum*, *Aerococcaceae*, *Spiroforme*, and *Bolteae* were abundant genera in our study. In a previous report, the abundance of *Bacteroides, Escherichia, Shigella*, and *Prevotella* was closely related to portal hypertension in cirrhosis patients^28^. Based on previous research, *Roseburia* and *Faecalibacterium prausnitzii* were regarded as good functioning strains. In this study, the normal control group revealed enrichment of *Roseburia* and *Faecalibacterium.* Taken together, the results indicate that the ML method using the composition of the macrobiome can be an efficient modality in the diagnosis of LD, especially alcoholic cirrhosis.

The investigators demonstrated that ALD is associated with disruptions in the gut microbiome. *Bacteroidetes* abundance was decreased in the heavy drinking control^29^. In another study, alcoholic ELE patients showed decreases in *Verrucomicrobia*, *Akkermansia*, and *Bacteroides*^30^. In this study, α and β diversity decreased according to disease progression. The *Proteobacteria* composition was increased in the cirrhosis group. The abundances of *Bacteroidaceae, Bacteroides*, *Equi*, *Butyricimonas*, *Erysipelotrichi*, and *Erysipelotrichales* were elevated in the ELE group. In addition, each group revealed different compositions of microbiota in our results. In ALD, the composition of the microbiota shows specific findings for each disease.

In our results, the diagnostic AUC of microbiota-based ML for Metabolic dysfunction-associated steatohepatitis (MASH) was 0.93 ± 0.11. In other reports, the decision-tree algorithm of the Canadian dataset diagnosed MASLD with 76% accuracy and an AUROC of 0.73^31^. Another study revealed that the AUROC of MASH was 0.83 to 0.88 in a large US population^32^. Considering that our data showed a higher diagnosis rate than other data using artificial intelligence utilizing clinical data, ML analysis of the intestinal microbiota showed a higher diagnostic rate. As the occurrence of MASH in Asia has increased in recent years, our results are considered a useful tool for the diagnosis of MASH in the future.

In this study, the CNN model trained with the 40 reduced feature dimensions had slightly higher sensitivity and specificity, showing good diagnostic classification power for predicting and identifying the disease groups. The reason might be that the architecture of the CNN used in this study is more suitable for capturing patterns within individual OUT sequences or understanding sequential relationships than other models such as RF or MLP. In a previous study, this gradient-boosting machine algorithm provided the best prediction of liver cancer risk in patients with virus infection^33^. Considering that ALD and MASLD are major public health problem and that approximately 50% of cirrhosis cases are related to ALD and MASLD in Asia^34^, it is necessary to develop various diagnostic technologies, including big data or ML techniques, that go beyond statistics.

We used the 16S rRNA method rather than the shotgun method because it is an inexpensive and easy method for rapid diagnosis and easy clinical use. In terms of diagnostic accuracy, the 16S rRNA-based ML strategy showed a high score and showed the basics of personal medicine in real time. Recently, multiomics-based analysis has been applied for the diagnosis, treatment, and prediction of various diseases^35^. The ML method using images, pathological tissues, and clinical results will be used in various fields of LD in the future. In South Korea, the prevalence of MASLD-related cirrhosis and liver cancer is low. Therefore, ML analysis for MASLD-related cirrhosis and liver cancer was not performed in this study.

MASLD is defined by using cardiometabolic markers. However, all patients did not perform liver biopsy for the diagnosis of MASH. To reduce selection bias, we used ELE group. Since MASLD diagnosis can be easily used clinically without a biopsy, it is expected to increase accessibility to the use of AI in the future. In South Korea, there are few cases of advanced liver disease (liver cirrhosis or HCC) associated with MASLD. We did not enrolled MASLD-related cirrhosis and HCC. Regarding elevated alpha diversities in control group compared with ELE group in MASLD, there are cases where the distribution of microbiotas changes and diversity increases due to liver disease.

## Conclusion

This study provides scientific evidence to support the excellent diagnostic accuracy of the microbiota for ALD and MASLD, suggesting holistic insight for further research. The CNN model trained with the 40 reduced feature dimensions had slightly higher sensitivity and specificity. This work developed an excellent microbiota-based ML strategy for the diagnosis of ALD and MASLD. Along with the development of personalized medicine, diagnostic technology using big data and genetic information will replace imaging or liver biopsy. The intestinal microbiota, which reflects an individual's health status, has a close relationship with LDs. Microbiota-based ML strategies can be used to diagnose ALD and MASLD to achieve personalized treatment and prevention of side effects. In the future, based on the microbiota-based ML strategy, we expect to develop a ML method for the treatment and follow-up.

## Methods

### Study design and participants

ALD was diagnosed on the results of alcohol history, liver biopsy, blood chemistry, or imaging study (ultrasound or computed tomography scan). The ALD group was subgrouped by control, ELE, cirrhosis, and liver cancer. Alcoholic ELE patients were defined if they had abnormal liver enzymes [aspartate aminotransferase (AST) ≥ 50 IU/L, AST/alanine aminotransferase (ALT) > 1.5, and AST and ALT < 400 IU/L] and excessive alcohol consumption (male > 60 g/day and female > 40 g/day) with last alcohol drink within 8 weeks of jaundice onset (bilirubin > 3 mg/dL). The control group was recruited from a medical check-up center. Patients meeting the following criteria were excluded: age > 70 years, hepatitis A, B, C, and E virus-related cirrhosis, HIV infection, Wilson’s disease, biliary obstruction, sepsis, drug-induced liver injury, autoimmune LD, history of high-dose steroid or antibiotics, presence of liver tumor or history of other cancer, or pregnancy.

MASLD was diagnosed on the definition as described in the consensus statement on new fatty liver disease nomenclature^36,37^. Cardiometabolic criteria including body mass index, fasting glucose, medication history, blood pressure, or cholesterol level were used for the diagnosis of MASLD. Normal control group did not have cardiometabolic criteria. Patients with elevated liver enzymes [AST or ALT ≥ 50 IU/L] or hepatitis on liver pathology were included in the ELE group. They did not drink excessive alcohol (male > 210 g/week and female > 140 g/week). Patients with autoimmune LD, alcohol use disorder, pancreatitis, hemochromatosis, viral LD, pregnancy, Wilson’s disease, drug-induced liver injury, and other cancers were excluded.

Cirrhosis was diagnosed based on the presence of complications (varix, ascites, and encephalopathy), blood tests, imaging findings, fibroscan, or pathological liver results. Liver cancer was diagnosed by two or more imaging tests, such as computer tomography, magnetic resonance imaging, angiography or contrast ultrasound. In addition, subjects taking drugs that affect the gut microbiota were excluded at enrollment. For the control group, we included healthy subjects who visited the center for a health check-up.

Baseline studies included family history, diet pattern, alcohol history, abdominal ultrasound and computed tomography scan, X-ray, electrocardiography, complete blood count, electrolytes, liver function test, viral markers, and Child‒Pugh score. Blood analysis was performed using standard methodologies. Serum biochemical parameters included AST, ALT, albumin, bilirubin, alkaline phosphatase (ALP), gamma glutamyl transpeptidase (GGT), blood urea nitrogen, creatinine, international normalized ratio, α-fetoprotein, carcinoembryonic antigen, prothrombin time, blood glucose, and total cholesterol. The levels of hepatitis A, B, and C and other virus markers were evaluated. Antinuclear antibody, antimitochondrial antibody, and antismooth muscle antibody tests were also performed.

This project followed the ethics of the 1975 Helsinki Declaration, as reflected by a prior approval by the Chuncheon Sacred Heart Hospital institutional review board for human research (2016–134). Informed consent was obtained from all participants. All authors had access to the study data and reviewed and approved the final manuscript.

### Validation cohort and data collection

Machine learning with internal validation was performed in 464 subjects, and 210 subjects were divided into an external validation group. For external validation, patient data collected from hospitals in different regions were used.

### Stool sample and processing

Sequencing was carried out according to the manufacturer’s instructions at Chunlab, Inc. (Seoul, Republic of Korea) with the Illumina MiSeq platform using reagent kit V3 in PE 250 bp mode. Microbiome taxonomic profiling was conducted with the EZBioCloud platform (ChunLab Inc., Republic of Korea) using the database version PKSSU4.0.

Human feces were stored at − 20 °C as soon as the patient received 2–3 g of feces using the kit (stool paper and stool box) and moved to − 80 °C within 1 day. Genomic DNA for metagenomic sequencing was extracted with a QIAamp stool kit (Qiagen, Hilden, Germany), and the library was prepared with a NEBNext Ultra II FS DNA Library Prep Kit for Illumina (New England BioLabs, Ipswish, MA, USA) according to the manufacturer’s directions. The quantification of libraries was checked using a Qubit dsDNA HS assay kit (Thermo Fisher Scientific, Waltham, MA, USA) and confirmed by quantitative polymerase chain reaction (qPCR) with a KAPA SYBR FAST qPCR Master Mix kit (Kapa Biosystems, Wilmington, MA, USA). The quality of the libraries was assessed on a Bioanalyzer 2100 (Agilent, Santa Clara, CA, USA) using a DNA 12,000 chip. All libraries were sequenced on the NovaSeq 6000 platform (Illumina, USA) with paired-end 150 bp reads.

The analysis was performed following our previous reference^38^. In brief, DNA was extracted with a QIAamp stool kit, and amplification of the V3-V4 region of the bacterial 16S rRNA gene was conducted using barcoded fusion primers. The forward fusion primer contained the p5 adapter, i5 index, and gene-specific primer 341F (5′-AATGATACGGCGACCACCGAGATCTACAC-XXXXXXXX-TCGTCGGCAGCGTCAGATGTG TATAAGAGACAG-CCTACGGGNGGCWGCAG3′; underlining indicates the target region primer and X indicates the barcode region), and the reverse fusion primer contained the p7 adapter, i7 index, and gene-specific primer 805R (5′-CAAGCAGAAGACGGCATACGAGATXXXXXXXXGTCTCGTGGGCTCGGAGATGTG TATAAGAGACAG-GACTACHVGGGTATCTAATCC-3′), which included sequencing adapters and dual-index barcodes of the Nextera XT kit (Illumina, San Diego, CA, USA). The amplification was performed in the C1000 touch thermal cycler PCR system (Bio-Rad Laboratories, Inc., Hercules, CA, USA) with the following conditions: initial denaturation of 3 min at 95 °C; followed by 25 cycles of denaturation at 95 °C for 30 s, annealing at 55 °C for 30 s, extension at 72 °C for 30 s and final extension at 72 °C for 5 min. Each amplified PCR product was confirmed with 1% agarose gel electrophoresis and visualized on a Gel Doc XR + imaging system (Bio-Rad Laboratories, Inc., USA). The amplified products were purified and size-selected by Agencourt AMPure XP beads (Beckman Coulter, Chaska, MN, USA). The library was constructed with pooled PCR products, and the quality of the library was assessed on a Bioanalyzer 2100 (Agilent, USA) using a DNA 12,000 chip and quantified by qPCR with a KAPA SYBR FAST qPCR Master Mix kit (Kapa Biosystems, USA).

### Sequence and statistical analysis

The sequencing data were processed using the Quantitative Insights Into Microbial Ecology (QIIME version 2). The low-quality sequence reads were removed following the criteria: (1) reads with a length of < 150 bp, (2) reads with an average Phred score of < 20, (3) reads containing ambiguous bases, and (4) reads containing mononucleotide repeats of > 8 bp. High-quality reads were clustered into 16S rRNA operational taxonomic units (OTUs) with ≥ 97% sequence homology^39^. The taxonomic classification of each OTU was performed with VSEARCH by comparing the representative sequence set against the SILVA reference database^40^.

After filtering samples with read counts greater than 500, the reads were grouped at the phylum level (using phyloseq), and the relative abundance was estimated at the phylum level by groups. There was a total of seven phyla. The taxa were sorted by abundance to improve the visualization and then plotted on box plots according to group and faceted by phylum using the raw counts. Many samples had a high number of Bacteroidetes, followed by Firmicutes and Proteobacteria. Most samples had low read counts for other phyla, with some outlying samples. To formally test for a difference in the phylum-level abundance, a multivariate test for differences in the overall composition between groups of samples was conducted by using the HMP package; herein, a Dirichlet-multinomial distribution is assumed for the data, and null hypothesis testing is conducted by testing for a difference in the location (mean distribution of each of the taxa) across groups accounting for the overdispersion in the count data.

Taxon abundance at the phylum, class, order, family, genus, and species levels was calculated and statistically compared among groups using the R stats package. Based on the tables generated in QIIME, alpha diversities, including Chao1, Simpson, and Shannon, were calculated. The significant differences in alpha diversity metrics were determined using the R package “Vegan”. To investigate the structural variation in microbial communities, beta diversity analysis was performed using UniFrac distance metrics 24 and was visualized via principal component analysis (PCA), principal coordinate analysis (PCOA), and nonmetric multidimensional scaling (NMDS). We checked the separation between other disease groups and normal group samples, suggesting some differences in the communities according to sample type. We tested whether the samples clustered beyond that expected by sampling variability using ADONIS.

The linear discriminant analysis (LDA) effect size (LEfSe) method based on the Kruskal‒Wallis test was conducted to identify significantly abundant taxa between different groups, where a linear discriminant analysis score > 2.0 was defined as the threshold for selecting the discriminative features. Quantitative data are expressed as the mean ± standard deviation (SD) unless otherwise stated. Comparisons were made utilizing analysis of variance (ANOVA), Kruskal‒Wallis test, general linear model analysis (repeated regression), paired sample t test, or independent-sample t test for continuous variables. The chi-square test or Fisher’s exact test was used for the comparison of groups. Data were analyzed with statistical software (SPSS, version 22.0, SPSS, Inc., Chicago, IL, USA), R stats package (www.r-project.org, Austria), and GraphPad Prism version 8.0 for Windows (GraphPad Software, San Diego, CA, USA). For all tests, *p* values < 0.05 were considered significant.

### Machine learning modeling for OTU feature reduction and classification

The abundance of microorganisms at the genus level was used as a feature. The abundance was normalized by applying a centered log ratio (clr) transformation. Three different nonsupervised ML algorithms for feature reduction were trained with the features of the normalized OTUs on the platform: independent component analysis (ICA), principal component analysis (PCA), and random projection (RP), considering 20, 40, 60, and 80 reduced features. For each of these numbers on the platform, four different supervised ML algorithms for classification were trained using the reduced features: support vector machine (SVM), random forest (RF), multilevel perceptron (MLP), and convolutional neural network (CNN) (Table 2 and Table S4). To evaluate the performance concerning model architectures for predictive classification and diagnostics of LDs, SVM, RF, MP, and CNN algorithms were constructed using various numbers of features reduced from the three nonsupervised ML algorithms. Additionally, four different supervised ML algorithms for classification were also trained with features having LDA score greater than 2.0. Data were assigned into training (70%) and testing internal validation (30%) datasets. The training performance of the different ML models was evaluated using Stratified ShuffleSplit tenfold cross-validation^41^, and the process was repeated 10 times. Hyperparameter tuning was automatically executed by caret, testing 60 different values for each hyperparameter.

**Table 2:**
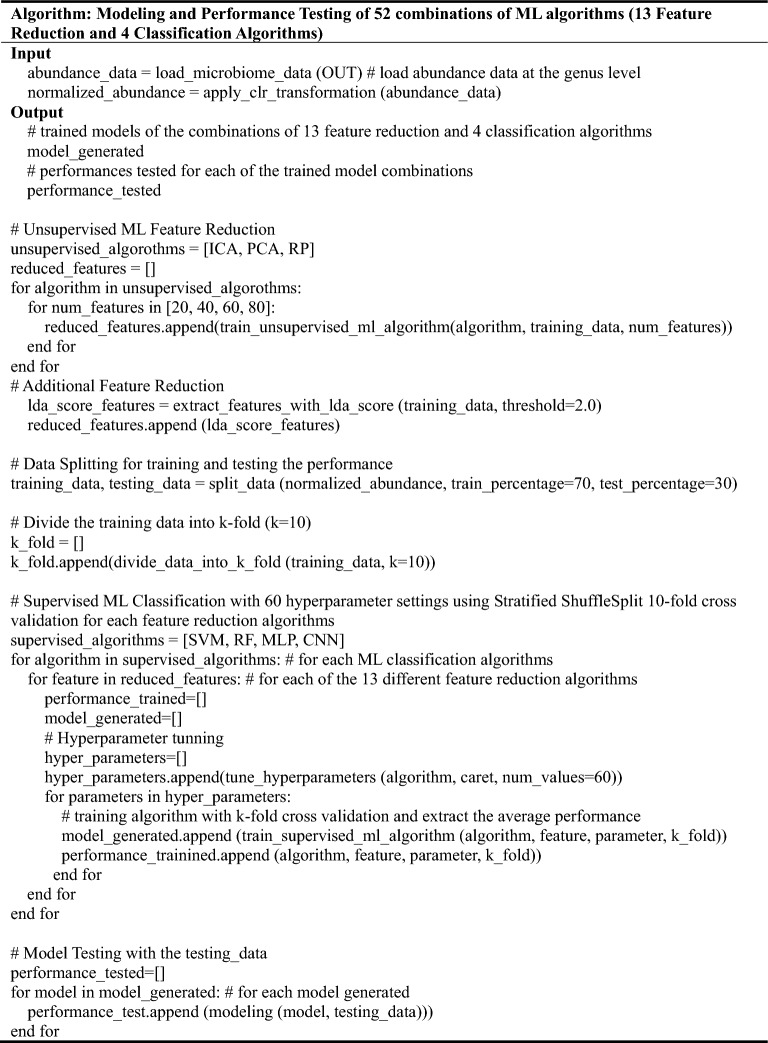
The modeling and performance testing process of 52 combinations of ML algorithms, 13 feature reduction and 4 classification algorithms.

A total of 52 combinations of MLs (ML for feature reduction with an ML for classification) for each of four different numbers (20, 40, 60 and 80) of reduced features were performed. The process was optimized using 60 hyperparameter variations and evaluated by Stratified ShuffleSplit tenfold cross validation. In the testing internal validation phase, the prediction performance of each combination of ML models was assessed using parameters including the area under the receiver operating characteristic curve (AUC), accuracy, recall, precision, and F1 score. Box plot representations of the AUC, accuracy, recall, precision, and F1 score values were generated using the ggplot2 package in R. The entire process was repeated for each pair of subgroups (7 ALD and 1 MASLD) within each group.

The computing machine we used for timestamped runs is on Ubuntu 18.04 and is equipped with an Intel Core i9-9820X CPU (10 cores), 64 GB memory, and an NVIDIA GTX 1080 Ti GPU. The scikit-learn Python library was utilized for ICA, PCA, RP, SVM, and RF. Additionally, the Keras Python library was employed for MLP and CNN.

### Supplementary Information

Supplementary Information.

## Data Availability

The data that supports the findings of this study are available in the supplementary material of this article.

## Abbreviations

LD: Liver disease
ALD: Alcohol-associated liver disease
MASLD: Metabolic dysfunction-associated steatotic liver disease
ML: Machine learning
AUC: Area under the receiver operating characteristic curve
AST: Aspartate aminotransferase
ALT: Alanine aminotransferase
PCA: Principal component analysis
PCOA: Principal coordinate analysis
NMDS: Nonmetric multidimensional scaling
LDA: Linear discriminant analysis
LEfSe: LDA effect size
ICA: Independent component analysis
PCA: Principal component analysis
RP: Random projection
SVM: Support vector machine
RF: Random forest
MLP: Multilevel perceptron
CNN: Convolutional neural network

## References

[1] Byass P (2014). The global burden of liver disease: A challenge for methods and for public health. BMC Med..

[2] Fan X, Shi Y, Han J, Song Y, Zhao J (2023). Beyond body weight: diversified presentation of MASLD in lean, overweight, and obese participants. J. Hepatol..

[3] Iruzubieta P, Santos-Laso A, Arias-Loste MT, Calleja JL, Crespo J (2023). Evaluation of metabolic dysfunction-Associated Steatotic liver disease (MASLD) terminology in different clinical settings. J. Hepatol..

[4] Singh SP, Panigrahi S, Mishra D, Khatua CR (2019). Alcohol-associated liver disease, not hepatitis B, is the major cause of cirrhosis in Asia. J. Hepatol..

[5] Li J, Nguyen MH (2020). Non-alcoholic fatty liver disease (NAFLD) in Asia-More efforts are needed. Liver Int..

[6] Wu T, Cooper SA, Shah VH (2022). Omics and AI advance biomarker discovery for liver disease. Nat. Med..

[7] Jiang P, Lai S, Wu S, Zhao XM, Chen WH (2020). Host DNA contents in fecal metagenomics as a biomarker for intestinal diseases and effective treatment. BMC Genom..

[8] Turnbaugh PJ (2007). The human microbiome project. Nature.

[9] Preidis GA, Versalovic J (2009). Targeting the human microbiome with antibiotics, probiotics, and prebiotics: Gastroenterology enters the metagenomics era. Gastroenterology.

[10] Ortigão R, Pimentel-Nunes P, Dinis-Ribeiro M, Libânio D (2020). Gastrointestinal microbiome—What we need to know in clinical practice. GE Port J. Gastroenterol..

[11] Haran JP, McCormick BA (2021). Aging, frailty, and the microbiome-how dysbiosis influences human aging and disease. Gastroenterology.

[12] Liu Y (2022). Early prediction of incident liver disease using conventional risk factors and gut-microbiome-augmented gradient boosting. Cell Metab..

[13] Hardjo M (2009). Suppression of carbon tetrachloride-induced liver fibrosis by transplantation of a clonal mesenchymal stem cell line derived from rat bone marrow. Cell Transplant..

[14] Jang YO (2014). Histological improvement following administration of autologous bone marrow-derived mesenchymal stem cells for alcoholic cirrhosis: A pilot study. Liver Int..

[15] Cresci GAM (2020). Is it time to consider gut microbiome readouts for precision diagnosis and treatment of alcoholic liver disease?. Hepatology.

[16] Mouzaki M, Loomba R (2020). An update on the role of the microbiome in non-alcoholic fatty liver disease pathogenesis, diagnosis, and treatment. Curr. Treat. Options Gastroenterol..

[17] Manandhar I (2021). Gut microbiome-based supervised machine learning for clinical diagnosis of inflammatory bowel diseases. Am. J. Physiol. Gastrointest Liver Physiol..

[18] Mossotto E (2017). Classification of paediatric inflammatory bowel disease using machine learning. Sci. Rep..

[19] Ulger Y, Delik A (2022). Artificial intelligence model with deep learning in nonalcoholic fatty liver disease diagnosis: Genetic based artificial neural networks. Nucleosides Nucleotides Nucleic Acids.

[20] Dana J (2022). Conventional and artificial intelligence-based imaging for biomarker discovery in chronic liver disease. Hepatol. Int..

[21] Lee HW, Sung JJY, Ahn SH (2021). Artificial intelligence in liver disease. J. Gastroenterol. Hepatol..

[22] Heshiki Y (2020). Predictable modulation of cancer treatment outcomes by the gut microbiota. Microbiome.

[23] Loomba R (2019). Gut microbiome-based metagenomic signature for non-invasive detection of advanced fibrosis in human nonalcoholic fatty liver disease. Cell Metab..

[24] Zitvogel L, Ma Y, Raoult D, Kroemer G, Gajewski TF (2018). The microbiome in cancer immunotherapy: Diagnostic tools and therapeutic strategies. Science.

[25] Pouncey AL, Scott AJ, Alexander JL, Marchesi J, Kinross J (2018). Gut microbiota, chemotherapy and the host: the influence of the gut microbiota on cancer treatment. Ecancermedicalscience.

[26] Li J (2014). An integrated catalog of reference genes in the human gut microbiome. Nat. Biotechnol..

[27] Kather JN (2019). Deep learning can predict microsatellite instability directly from histology in gastrointestinal cancer. Nat. Med..

[28] Gedgaudas R (2022). Circulating microbiome in patients with portal hypertension. Gut Microbes.

[29] Smirnova E (2020). Fecal microbiome distinguishes alcohol consumption from alcoholic hepatitis but does not discriminate disease severity. Hepatology.

[30] Lang S (2020). Changes in the fecal bacterial microbiota associated with disease severity in alcoholic hepatitis patients. Gut Microbes.

[31] Perveen S, Shahbaz M, Keshavjee K, Guergachi A (2018). A systematic machine learning based approach for the diagnosis of non-alcoholic fatty liver disease risk and progression. Sci. Rep..

[32] Fialoke S, Malarstig A, Miller MR, Dumitriu A (2018). Application of machine learning methods to predict non-alcoholic steatohepatitis (NASH) in non-alcoholic fatty liver (NAFL) patients. AMIA Annu. Symp. Proc..

[33] Kim HY (2022). An artificial intelligence model to predict hepatocellular carcinoma risk in Korean and Caucasian patients with chronic hepatitis B. J. Hepatol..

[34] Golabi P (2021). Burden of non-alcoholic fatty liver disease in Asia, the Middle East and North Africa: Data from Global Burden of Disease 2009–2019. J Hepatol.

[35] Sharma N (2022). Bile multi-omics analysis classifies lipid species and microbial peptides predictive of carcinoma of gallbladder. Hepatology.

[36] Fukunaga S, Mukasa M, Nakano D, Tsutsumi T, Kawaguchi T (2023). Changing from NAFLD to MASLD: similar cumulative incidence of reflux Esophagitis between NAFLD and MASLD. Clin. Mol. Hepatol..

[37] Rinella ME (2023). A multisociety Delphi consensus statement on new fatty liver disease nomenclature. Hepatology.

[38] Song HS (2020). Microbial niches in raw ingredients determine microbial community assembly during kimchi fermentation. Food Chem..

[39] Nguyen NP, Warnow T, Pop M, White B (2016). A perspective on 16S rRNA operational taxonomic unit clustering using sequence similarity. NPJ Biofilms Microbiomes.

[40] Rognes T, Flouri T, Nichols B, Quince C, Mahe F (2016). VSEARCH: A versatile open source tool for metagenomics. PeerJ.

[41] Pedregosa F, Varoquaux G, Gramfort A, Michel V, Thirion B, Grisel O, Blondel M, Prettenhofer P, Weiss R, Dubourg V, Vanderplas J, Passos A, Cournapeau D, Brucher M, Perrot M, Duchesnay E (2011). Scikit-learn: Machine learning in python. J. Mach. Learn. Res..

