# Supplementary material for: Gut microbiota-based machine-learning signature for the diagnosis of alcohol-associated and metabolic dysfunction-associated steatotic liver disease
Source: Sci Rep. 2024 Jul 12;14:16122. doi: 10.1038/s41598-024-60768-2 (PMC11245548; doi:10.1038/s41598-024-60768-2)
Supplement: Supplementary file 1 — Supplementary Information. [file 41598_2024_60768_MOESM1_ESM.docx]

**Supplementary file**

**Supplement Table1. Baseline characteristics**

| **Training and test set** | | | | | | |
| --- | --- | --- | --- | --- | --- | --- |
|  | Alcoholic liver disease | | | | MASLD | |
| Mean (SD) | Normal control (n=74) | ELE  (n=60) | Cirrhosis (n=81) | HCC  (n=48) | Normal control (n=77) | ELE  (n=124) |
| Male (n, [%]) | 36 (48) | 39 (75) | 51 (64) | 29 (63) | 39 (51) | 62 (50) |
| Age (year) | 61 (7) | 51 (12) | 54 (11) | 62 (13) | 63 (8) | 57 (12) |
| Alcohol drinking (g/wk) | 50 (24) | 260 (40) | 200 (55) | 120 (89) | 30 (150 | 40 (28) |
| AST (IU/L) | 25 (10) | 85 (96) | 111 (152) | 103 (145) | 24 (8) | 47 (61) |
| ALT (IU/L) | 25 (16) | 61 (46) | 39 (44) | 63 (100) | 24 (17) | 54 (38) |
| γGT (IU/L) | 44 (49) | 325 (474) | 437 (775) | 235 (252) | 32 (36) | 57 (53) |
| Creatinine (mg/dL) | 0.9 (0.8) | 0.9 (0.7) | 0.8 (0.7) | 0.8 (0.9) | 0.7 (0.7) | 0.9 (1.0) |
| Cholesterol (mg/dL) | 180 (36) | 170 (53) | 137 (54) | 128 (53) | 167 (37) | 178 (38) |
| BMI | 25 (3) | 24 (4) | 24 (3) | 24 (4) | 24 (4) | 25 (4) |
| **Validation set** | | | | | | |
|  | Normal control (n=26) | ELE  (n=24) | Cirrhosis (n=59) | HCC  (n=17) | Normal control (n=22) | ELE  (n=62) |
| Male (n, [%]) | 14 (54) | 18 (75) | 34 (58) | 10 (59) | 12 (55) | 30 (48) |
| Age (year) | 57 (11) | 54 (9) | 56 (10) | 63 (8) | 65 (11) | 59 (10) |
| Alcohol drinking (g/wk) | 42 (18) | 274 (34) | 220 (58) | 156 (80) | 15 (10) | 28 (17) |
| AST (IU/L) | 37 (10) | 85 (96) | 130 (122) | 112 (72) | 30 (9) | 55 (41) |
| ALT (IU/L) | 30 (16) | 71 (40) | 49 (43) | 53 (92) | 30 (17) | 54 (38) |
| γGT (IU/L) | 44 (49) | 325 (474) | 437 (775) | 235 (252) | 32 (36) | 59 (50) |
| Creatinine (mg/dL) | 0.9 (0.8) | 0.9 (0.7) | 0.9 (0.7) | 0.9 (0.9) | 0.8 (0.7) | 0.8 (1.0) |
| Cholesterol (mg/dL) | 177 (30) | 179 (53) | 140 (52) | 130 (59) | 167 (37) | 199 (30) |
| BMI | 23 (3) | 22 (4) | 23 (3) | 24 (4) | 23 (4) | 24 (4) |

ELE, elevated liver enzyme; SD, standard deviation; n, number; MASLD, metabolic dysfunction-associated steatotic liver disease; BMI, body mass index; AST, aspartate aminotransferase; ALT, alanine aminotransferase; γGT, gamma glutamyl transpeptidase

**Supplement Table 2.** Taxonomical features of ALD groups with a LDA score > 2.0

| Feature-species or genus | Group | LDA score | p-value |
| --- | --- | --- | --- |
| *Bacteria.Proteobacteria* | HCC | 4.98029 | 0.000468 |
| *Bacteria.Actinobacteria.Actinobacteria.Bifidobacteriales.Bifidobacteriaceae.Bifidobacterium.bifidum* |  | 2.936097 | 0.002065 |
| *Bacteria.Bacteroidetes.Bacteroidia.Bacteroidales._Paraprevotellaceae_* |  | 3.095678 | 0.003835 |
| *Bacteria.Firmicutes.Bacilli.Bacillales.Bacillaceae.Bacillus* |  | 3.340567 | 0.035208 |
| *Bacteria.Firmicutes.Bacilli.Lactobacillales.Lactobacillaceae* |  | 3.352703 | 2.50E-07 |
| *Bacteria.Firmicutes.Bacilli.Lactobacillales.Lactobacillaceae.Lactobacillus.hamsteri* |  | 4.331152 | 5.56E-08 |
| *Bacteria.Firmicutes.Bacilli.Lactobacillales.Streptococcaceae* |  | 4.053847 | 2.32E-09 |
| *Bacteria.Firmicutes.Bacilli.Lactobacillales.Streptococcaceae.Streptococcus* |  | 3.926088 | 1.62E-10 |
| *Bacteria.Proteobacteria.Betaproteobacteria* |  | 3.381573 | 0.006841 |
| *Bacteria.Proteobacteria.Gammaproteobacteria* |  | 4.976148 | 3.25E-05 |
| *Bacteria.Proteobacteria.Gammaproteobacteria.Enterobacteriales* |  | 4.950695 | 0.00016 |
| *Bacteria.Proteobacteria.Gammaproteobacteria.Enterobacteriales.Enterobacteriaceae* |  | 4.948545 | 0.00016 |
| *Bacteria.Actinobacteria.Coriobacteriia* | Cirrhosis | 3.846128 | 0.013704 |
| *Bacteria.Actinobacteria.Coriobacteriia.Coriobacteriales* |  | 3.618718 | 0.013704 |
| *Bacteria.Actinobacteria.Coriobacteriia.Coriobacteriales.Coriobacteriaceae.Collinsella* |  | 3.691356 | 0.037725 |
| *Bacteria.Actinobacteria.Coriobacteriia.Coriobacteriales.Coriobacteriaceae.Collinsella.* |  | 3.67206 | 0.037725 |
| *Bacteria.Firmicutes* |  | 3.582376 | 4.07E-07 |
| *Bacteria.Firmicutes.Bacilli.Lactobacillales.Aerococcaceae* |  | 3.525972 | 1.36E-06 |
| *Bacteria.Firmicutes.Bacilli.Lactobacillales.Aerococcaceae.Granulicatella* |  | 3.786091 | 4.68E-06 |
| *Bacteria.Firmicutes.Bacilli.Lactobacillales.Aerococcaceae.Granulicatella.balaenopterae* |  | 3.742738 | 4.68E-06 |
| *Bacteria.Firmicutes.Bacilli.Lactobacillales.Lactobacillaceae.Lactobacillus.paraplantarum* |  | 3.527659 | 0.000141 |
| *Bacteria.Firmicutes.Clostridia.Clostridiales.Lachnospiraceae.Clostridium.bolteae* |  | 3.114784 | 0.000437 |
| *Bacteria.Firmicutes.Clostridia.Clostridiales.Veillonellaceae.Anaerosinus* |  | 4.522358 | 1.18E-08 |
| *Bacteria.Firmicutes.Clostridia.Clostridiales.Veillonellaceae.Anaerosinus.glycerini* |  | 4.522358 | 1.18E-08 |
| *Bacteria.Firmicutes.Erysipelotrichi.Erysipelotrichales.Erysipelotrichaceae.Clostridium* |  | 3.016167 | 3.03E-08 |
| *Bacteria.Firmicutes.Erysipelotrichi.Erysipelotrichales.Erysipelotrichaceae.Clostridium.spiroforme* |  | 3.463909 | 0.002096 |
| *Bacteria.Bacteroidetes.Bacteroidia.Bacteroidales._Odoribacteraceae_.Butyricimonas* | ELE | 3.604114 | 0.000251 |
| *Bacteria.Bacteroidetes.Bacteroidia.Bacteroidales.Bacteroidaceae* |  | 4.86534 | 0.020975 |
| *Bacteria.Bacteroidetes.Bacteroidia.Bacteroidales.Bacteroidaceae.Bacteroides* |  | 4.829277 | 0.012311 |
| *Bacteria.Firmicutes.Bacilli.Lactobacillales.Streptococcaceae.Streptococcus.equi* |  | 3.741788 | 0.000142 |
| *Bacteria.Firmicutes.Erysipelotrichi* |  | 3.15235 | 0.008669 |
| *Bacteria.Firmicutes.Erysipelotrichi.Erysipelotrichales* |  | 3.15235 | 0.008669 |
| *Bacteria.* | Control | 3.035608 | 0.003041 |
| *Bacteria.Bacteroidetes.Bacteroidia.Bacteroidales* |  | 3.229782 | 0.002949 |
| *Bacteria.Bacteroidetes.Bacteroidia.Bacteroidales._Odoribacteraceae_* |  | 3.62275 | 6.30E-06 |
| *Bacteria.Bacteroidetes.Bacteroidia.Bacteroidales._Odoribacteraceae_.Odoribacter* |  | 3.539695 | 2.97E-05 |
| *Bacteria.Bacteroidetes.Bacteroidia.Bacteroidales._Paraprevotellaceae_.Paraprevotella* |  | 3.397106 | 1.60E-06 |
| *Bacteria.Bacteroidetes.Bacteroidia.Bacteroidales.Rikenellaceae.Alistipes* |  | 3.089576 | 1.18E-05 |
| *Bacteria.Bacteroidetes.Bacteroidia.Bacteroidales.Rikenellaceae.Alistipes.finegoldii* |  | 3.258005 | 3.20E-07 |
| *Bacteria.Bacteroidetes.Bacteroidia.Bacteroidales.Rikenellaceae.Alistipes.putredinis* |  | 3.853025 | 1.33E-05 |
| *Bacteria.Bacteroidetes.Bacteroidia.Bacteroidales.S24_7* |  | 3.333313 | 2.80E-07 |
| *Bacteria.Firmicutes.Clostridia.Clostridiales* |  | 3.201171 | 4.19E-05 |
| *Bacteria.Firmicutes.Clostridia.Clostridiales.Lachnospiraceae* |  | 3.762187 | 8.06E-12 |
| *Bacteria.Firmicutes.Clostridia.Clostridiales.Lachnospiraceae.Clostridium* |  | 3.033402 | 7.11E-05 |
| *Bacteria.Firmicutes.Clostridia.Clostridiales.Lachnospiraceae.Roseburia* |  | 3.760318 | 1.33E-12 |
| *Bacteria.Firmicutes.Clostridia.Clostridiales.Lachnospiraceae.Roseburia.inulinivorans* |  | 3.757443 | 1.33E-12 |
| *Bacteria.Firmicutes.Clostridia.Clostridiales.Lachnospiraceae.Ruminococcus.lactaris* |  | 3.234862 | 5.18E-09 |
| *Bacteria.Firmicutes.Clostridia.Clostridiales.Ruminococcaceae* |  | 3.60827 | 1.04E-07 |
| *Bacteria.Firmicutes.Clostridia.Clostridiales.Ruminococcaceae.Clostridium.islandicum* |  | 2.975613 | 0.000145 |
| *Bacteria.Firmicutes.Clostridia.Clostridiales.Ruminococcaceae.Faecalibacterium* |  | 3.484471 | 3.57E-06 |
| *Bacteria.Firmicutes.Clostridia.Clostridiales.Ruminococcaceae.Gemmiger* |  | 3.733309 | 4.28E-16 |
| *Bacteria.Firmicutes.Clostridia.Clostridiales.Ruminococcaceae.Gemmiger.formicilis* |  | 3.732198 | 4.28E-16 |
| *Bacteria.Firmicutes.Erysipelotrichi.Erysipelotrichales.Erysipelotrichaceae* |  | 2.835949 | 0.001467 |
| *Bacteria.Proteobacteria.Betaproteobacteria.Burkholderiales* |  | 3.479398 | 0.006892 |
| *Bacteria.Proteobacteria.Deltaproteobacteria* |  | 3.366494 | 0.000316 |
| *Bacteria.Proteobacteria.Deltaproteobacteria.Desulfovibrionales.Desulfovibrionaceae* |  | 3.224232 | 6.34E-05 |

ELE, elevated liver enzyme

**Supplement Table 3.** Taxonomical features of MASLD groups with a LDA score>2.0

| Feature | Group | LDA score | pvalue |
| --- | --- | --- | --- |
| *Bacteria.Actinobacteria* | ELE | 3.442449 | 0.01728 |
| *Bacteria.Actinobacteria.Actinobacteria* |  | 3.348942 | 0.033073 |
| *Bacteria.Actinobacteria.Actinobacteria.Bifidobacteriales* |  | 3.172292 | 0.043287 |
| *Bacteria.Actinobacteria.Actinobacteria.Bifidobacteriales.Bifidobacteriaceae* |  | 3.172292 | 0.043287 |
| *Bacteria.Actinobacteria.Coriobacteriia* |  | 2.948305 | 0.001026 |
| *Bacteria.Actinobacteria.Coriobacteriia.Coriobacteriales* |  | 3.00883 | 0.001026 |
| *Bacteria.Actinobacteria.Coriobacteriia.Coriobacteriales.Coriobacteriaceae.Collinsella* |  | 2.819818 | 0.002107 |
| *Bacteria.Actinobacteria.Coriobacteriia.Coriobacteriales.Coriobacteriaceae.Collinsella.* |  | 2.830713 | 0.002107 |
| *Bacteria.Bacteroidetes.Bacteroidia.Bacteroidales.Bacteroidaceae.Bacteroides.coprophilus* |  | 3.086635 | 0.043848 |
| *Bacteria.Bacteroidetes.Bacteroidia.Bacteroidales.Rikenellaceae.Alistipes.indistinctus* |  | 2.650614 | 0.027185 |
| *Bacteria.Firmicutes* |  | 3.819271 | 0.00023 |
| *Bacteria.Firmicutes.Bacilli* |  | 4.283469 | 0.007615 |
| *Bacteria.Firmicutes.Bacilli.Lactobacillales.Aerococcaceae* |  | 2.304982 | 0.000132 |
| *Bacteria.Firmicutes.Bacilli.Lactobacillales.Aerococcaceae.Granulicatella* |  | 2.769129 | 0.000288 |
| *Bacteria.Firmicutes.Bacilli.Lactobacillales.Aerococcaceae.Granulicatella.balaenopterae* |  | 2.787935 | 0.000288 |
| *Bacteria.Firmicutes.Bacilli.Lactobacillales.Lactobacillaceae* |  | 2.125523 | 0.009002 |
| *Bacteria.Firmicutes.Bacilli.Lactobacillales.Leuconostocaceae.Weissella* |  | 2.519355 | 0.004535 |
| *Bacteria.Firmicutes.Bacilli.Lactobacillales.Leuconostocaceae.Weissella.hellenica* |  | 2.516458 | 0.004535 |
| *Bacteria.Firmicutes.Bacilli.Lactobacillales.Streptococcaceae* |  | 3.912715 | 5.29E-06 |
| *Bacteria.Firmicutes.Bacilli.Lactobacillales.Streptococcaceae.Streptococcus* |  | 3.263277 | 2.50E-05 |
| *Bacteria.Firmicutes.Bacilli.Lactobacillales.Streptococcaceae.Streptococcus.equi* |  | 3.804053 | 4.92E-06 |
| *Bacteria.Firmicutes.Clostridia.Clostridiales.Lachnospiraceae.Clostridium.bolteae* |  | 2.614449 | 0.045096 |
| *Bacteria.Firmicutes.Clostridia.Clostridiales.Ruminococcaceae.Butyricicoccus* |  | 2.521415 | 0.013539 |
| *Bacteria.Firmicutes.Clostridia.Clostridiales.Ruminococcaceae.Butyricicoccus.pullicaecorum* |  | 2.528662 | 0.013539 |
| *Bacteria.Firmicutes.Clostridia.Clostridiales.Ruminococcaceae.Subdoligranulum* |  | 2.713045 | 0.01038 |
| *Bacteria.Firmicutes.Clostridia.Clostridiales.Ruminococcaceae.Subdoligranulum.variabile* |  | 2.708402 | 0.01038 |
| *Bacteria.Firmicutes.Clostridia.Clostridiales.Veillonellaceae.Anaerosinus* |  | 3.906862 | 0.009653 |
| *Bacteria.Firmicutes.Clostridia.Clostridiales.Veillonellaceae.Anaerosinus.glycerini* |  | 3.906862 | 0.009653 |
| *Bacteria.Firmicutes.Clostridia.Clostridiales.Veillonellaceae.Succinispira* |  | 4.032886 | 0.045854 |
| *Bacteria.Firmicutes.Clostridia.Clostridiales.Veillonellaceae.Succinispira.mobilis* |  | 4.032886 | 0.045854 |
| *Bacteria.Firmicutes.Erysipelotrichi* |  | 3.588245 | 0.007313 |
| *Bacteria.Firmicutes.Erysipelotrichi.Erysipelotrichales* |  | 3.588245 | 0.007313 |
| *Bacteria.Firmicutes.Erysipelotrichi.Erysipelotrichales.Erysipelotrichaceae._Eubacterium_.biforme* |  | 3.080602 | 0.004719 |
| *Bacteria.Firmicutes.Erysipelotrichi.Erysipelotrichales.Erysipelotrichaceae.Catenibacterium* |  | 3.282876 | 0.017044 |
| *Bacteria.Firmicutes.Erysipelotrichi.Erysipelotrichales.Erysipelotrichaceae.Catenibacterium.mitsuokai* |  | 3.302578 | 0.017044 |
| *Bacteria.Firmicutes.Erysipelotrichi.Erysipelotrichales.Erysipelotrichaceae.Holdemania* |  | 2.137383 | 0.048915 |
| *Bacteria.TM7* |  | 3.294099 | 0.023312 |
| *Bacteria.TM7.TM7_3* |  | 3.305751 | 0.023312 |
| *Bacteria.Bacteroidetes.Bacteroidia* | Control | 4.834159 | 0.000115 |
| *Bacteria.Bacteroidetes.Bacteroidia.Bacteroidales.Bacteroidaceae* |  | 4.68532 | 0.005846 |
| *Bacteria.Bacteroidetes.Bacteroidia.Bacteroidales.Bacteroidaceae.Bacteroides* |  | 4.52123 | 0.011842 |
| *Bacteria.Firmicutes.Bacilli.Lactobacillales.Lactobacillaceae.Lactobacillus.hamsteri* |  | 2.134249 | 0.034482 |
| *Bacteria.Firmicutes.Erysipelotrichi.Erysipelotrichales.Erysipelotrichaceae.Clostridium* |  | 2.350757 | 0.036871 |
| *Bacteria.Proteobacteria.Betaproteobacteria* |  | 2.493839 | 0.022642 |
| *Bacteria.Proteobacteria.Deltaproteobacteria.Desulfovibrionales* |  | 2.243218 | 0.027175 |
| *Bacteria.Proteobacteria.Deltaproteobacteria.Desulfovibrionales.Desulfovibrionaceae.Desulfovibrio.D168* |  | 2.434182 | 0.014964 |

ELE, elevated liver enzyme;

**Supplement Table 4.** Performance measures of CNN model using 20, 40, 60, 80 feature dimensions reduced from PCA model for ALD datasets

| **Group 1** | **Group 2** | Performance | | | | | |
| --- | --- | --- | --- | --- | --- | --- | --- |
|  |  | No. of Features | AUC | ACC | Recall | Precision | F1 |
| Control | ELE | 20 | 0.92±0.08 | 0.93±0.08 | 0.94±0.08 | 0.89±0.13 | 0.91±0.10 |
|  |  | 40 | 0.95±0.08 | 0.95±0.09 | 0.95±0.09 | 0.94±0.12 | 0.94±0.10 |
|  |  | 60 | 0.94±0.09 | 0.94±0.08 | 0.96±0.07 | 0.92±0.13 | 0.93±0.10 |
|  |  | 80 | 0.94±0.10 | 0.95±0.10 | 0.96±0.09 | 0.93±0.11 | 0.94±0.12 |
|  | Cirrhosis | 20 | 0.95±0.08 | 0.95±0.09 | 0.96±0.06 | 0.94±0.14 | 0.95±0.1 |
|  |  | 40 | 0.96±0.07 | 0.96±0.07 | 0.98±0.04 | 0.95±0.12 | 0.96±0.08 |
|  |  | 60 | 0.97±0.06 | 0.97±0.07 | 0.98±0.03 | 0.96±0.11 | 0.97±0.07 |
|  |  | 80 | 0.96±0.07 | 0.96±0.08 | 0.98±0.05 | 0.96±0.11 | 0.96±0.08 |
|  | HCC | 20 | 0.97±0.07 | 0.98±0.06 | 0.98±0.06 | 0.96±0.11 | 0.97±0.08 |
|  |  | 40 | 0.96±0.08 | 0.97±0.07 | 0.96±0.09 | 0.96±0.13 | 0.95±0.11 |
|  |  | 60 | 0.96±0.09 | 0.97±0.07 | 0.96±0.10 | 0.94±0.15 | 0.95±0.12 |
|  |  | 80 | 0.96±0.08 | 0.97±0.07 | 0.97±0.05 | 0.95±0.13 | 0.95±0.11 |
| Normal+  ELE | Cirrhosis+HCC | 20 | 0.96±0.07 | 0.96±0.07 | 0.97±0.06 | 0.95±0.08 | 0.96±0.07 |
|  |  | 40 | 0.96±0.07 | 0.97±0.07 | 0.97±0.06 | 0.96±0.10 | 0.96±0.08 |
|  |  | 60 | 0.96±0.07 | 0.96±0.07 | 0.96±0.07 | 0.96±0.10 | 0.96±0.08 |
|  |  | 80 | 0.95±0.07 | 0.96±0.07 | 0.96±0.06 | 0.95±0.10 | 0.95±0.09 |
| ELE | Cirrhosis | 20 | 0.93±0.11 | 0.94±0.10 | 0.95±0.09 | 0.94±0.09 | 0.94±0.09 |
|  |  | 40 | 0.94±0.12 | 0.95±0.11 | 0.96±0.11 | 0.95±0.09 | 0.95±0.09 |
|  |  | 60 | 0.93±0.13 | 0.94±0.12 | 0.94±0.12 | 0.96±0.08 | 0.95±0.10 |
|  |  | 80 | 0.93±0.14 | 0.93±0.14 | 0.94±0.13 | 0.97±0.05 | 0.95±0.09 |
|  | HCC | 20 | 0.95±0.08 | 0.96±0.07 | 0.99±0.03 | 0.94±0.12 | 0.95±0.09 |
|  |  | 40 | 0.96±0.07 | 0.97±0.07 | 0.99±0.03 | 0.96±0.09 | 0.96±0.08 |
|  |  | 60 | 0.97±0.06 | 0.98±0.06 | 0.98±0.08 | 0.96±0.09 | 0.97±0.07 |
|  |  | 80 | 0.97±0.05 | 0.98±0.05 | 0.99±0.02 | 0.96±0.07 | 0.97±0.06 |
| Cirrhosis | HCC | 20 | 0.93±0.12 | 0.94±0.12 | 0.95±0.16 | 0.9±0.17 | 0.91±0.17 |
|  |  | 40 | 0.93±0.11 | 0.94±0.10 | 0.95±0.11 | 0.91±0.16 | 0.92±0.14 |
|  |  | 60 | 0.93±0.12 | 0.94±0.11 | 0.96±0.12 | 0.88±0.20 | 0.91±0.16 |
|  |  | 80 | 0.92±0.13 | 0.94±0.11 | 0.95±0.12 | 0.88±0.22 | 0.90±0.18 |

ELE, elevated liver enzyme; HCC, hepatocellular carcinoma; ICA, independent component analysis; PCA, principal component analysis; RP, random projection; SVM, support vector machine; RF, random forest; MLP, multilevel perceptron; CNN, convolutional neural network

Forty reduced feature dimensions are considered from each of ICA, PCA, and RP.

**Supplement Table 5.** Performance measures of CNN model using 20, 40, 60, 80 feature dimensions reduced from PCA model for MASLD datasets

| **Group 1** | **Group 2** | **Performance** | | | | | | |
| --- | --- | --- | --- | --- | --- | --- | --- | --- |
|  |  | Number of features | AUC | ACC | Recall | Precision | F1 | |
| Normal | ELE | 20 | 0.91±0.12 | 0.92±0.11 | 0.93±0.09 | 0.94±0.10 | 0.93±0.09 | |
|  |  | 40 | 0.93±0.11 | 0.94±0.11 | 0.95±0.09 | 0.95±0.05 | 0.95±0.10 | |
|  |  | 60 | 0.94±0.13 | 0.95±0.12 | 0.95±0.10 | 0.97±0.04 | 0.96±0.09 | |
|  |  | 80 | 0.95±0.10 | 0.96±0.09 | 0.96±0.09 | 0.98±0.05 | 0.97±0.07 |  |

**Supplement Table 6.** The CNN architecture used for 40 reduced feature dimensions in this study

|  | Layer Type | Activation Unit | Number of Filters | Kernel Size | Number of Stride | Padding | Output Size | Number of Parameters |
| --- | --- | --- | --- | --- | --- | --- | --- | --- |
| 1 | Input |  |  |  |  |  | 40 X 1 |  |
| 2 | Conv1D | Relu | 256 | 3 | 1 | VALID | 38 X 256 | 1024 |
| 3 | Conv1D | Relu | 128 | 3 | 1 | VALID | 36 X 128 | 98432 |
| 4 | Dropout (0.5) |  |  |  |  |  | 36 X 128 | 0 |
| 5 | MaxPooling1D | - | 2 | - | - | - | 18 X 128 | 0 |
| 6 | Flaten |  |  |  |  |  | 2304 | 0 |
| 7 | Fully connected | Relu |  |  |  |  | 64 | 147520 |
| 8 | Output | Sigmoid |  |  |  |  | 1 | 65 |


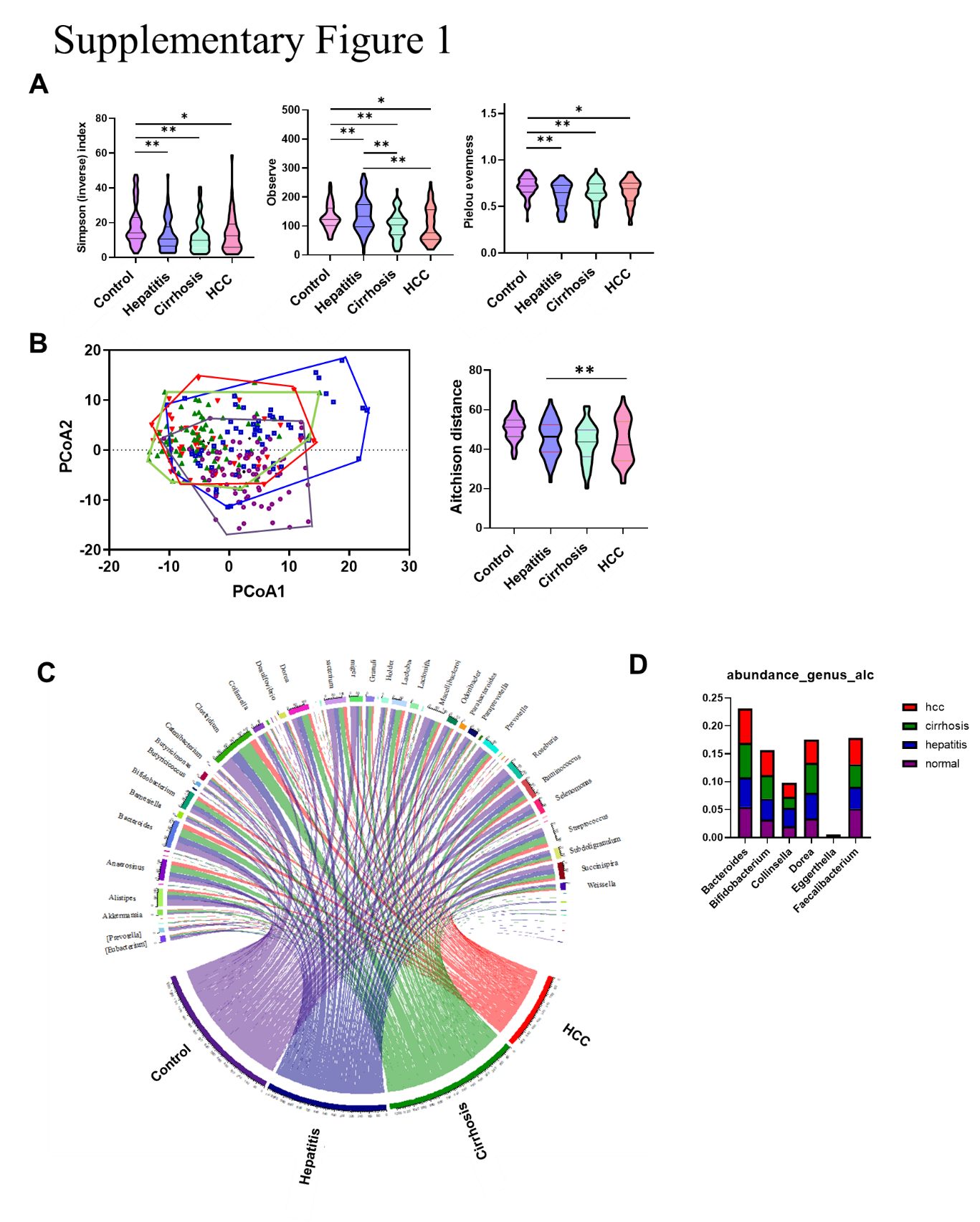


**Supplementary Figure 1.** Differences in ALD group. α diversity (A). β diversity (B). The inter-microbiome and disease correlation networks (C). Abundance of genus (D).


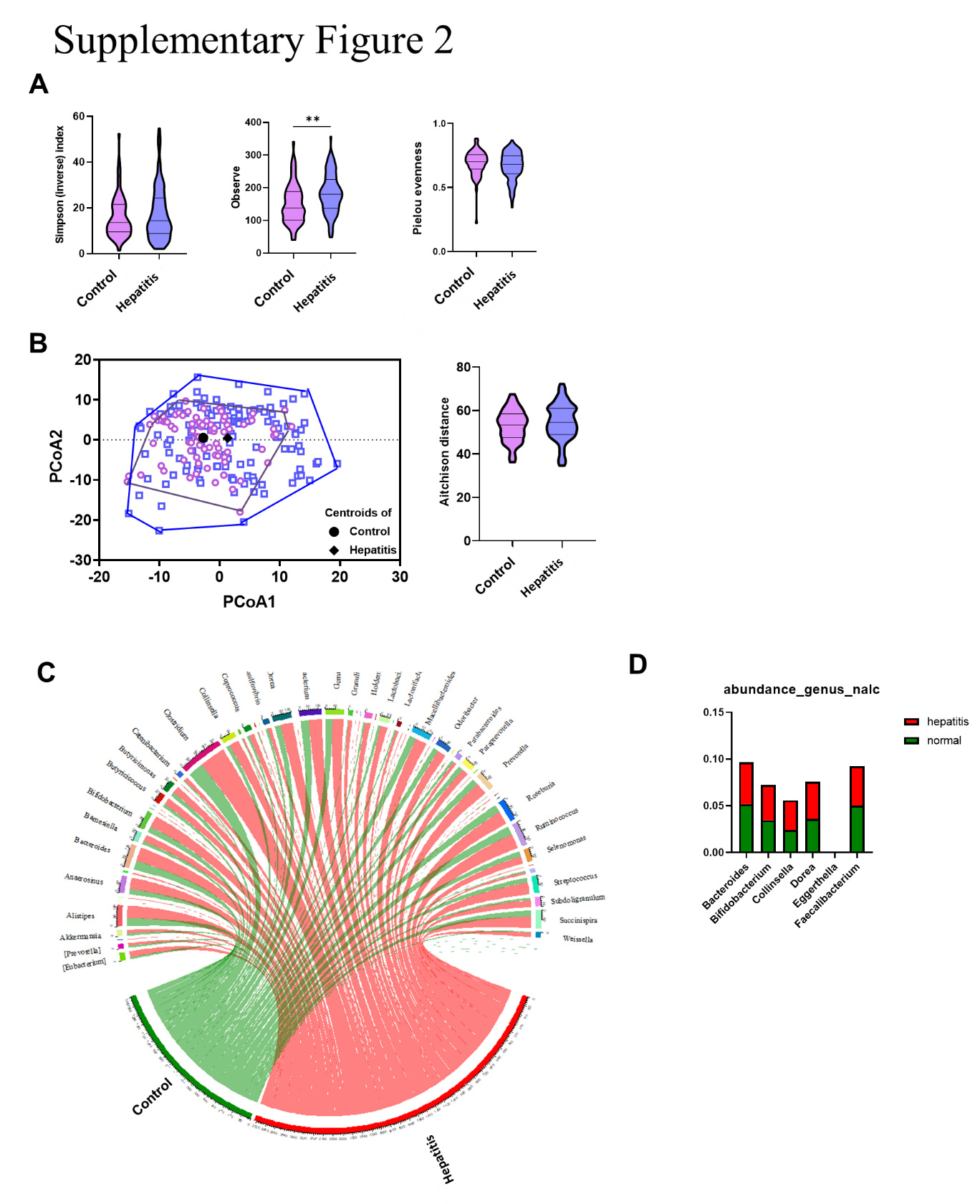


**Supplementary Figure 2.** Differences in MASLD group. α diversity (A). β diversity (B). The inter-microbiome and disease correlation networks (C). Abundance of genus (D).

**Supplemenrary Figure 3**

**
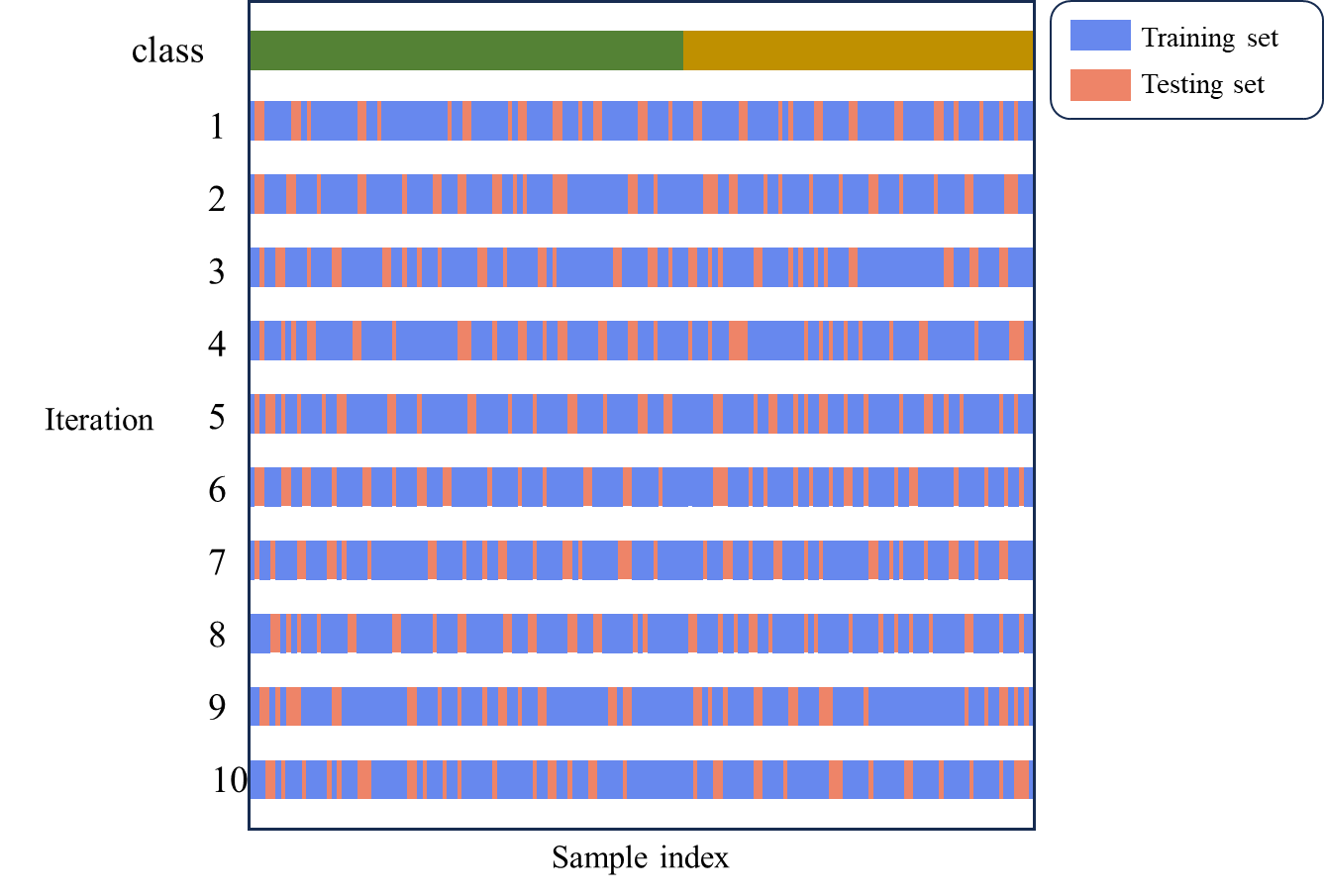
**

Supplementary Figure 3. A visualization of the training and testing data subsets generated in this study for each of the 10 iterations, adopting the Stratified ShuffleSplit 10-fold cross-validation technique.
